# Topical co‐administration of zoledronate with recombinant human bone morphogenetic protein-2 can induce and maintain bone formation in the bone marrow environment

**DOI:** 10.1186/s12891-021-03971-w

**Published:** 2021-01-20

**Authors:** Hideki Ueyama, Yoichi Ohta, Yuuki Imai, Akinobu Suzuki, Ryo Sugama, Yukihide Minoda, Kunio Takaoka, Hiroaki Nakamura

**Affiliations:** 1 Department of Orthopaedic Surgery, Osaka City University Graduate School of Medicine, 1-4-3 Asahimachi, Abeno-ku, 545-8585 Osaka, Japan; 2 Division of Integrative Pathophysiology, Proteo-Science Center, Graduate School of Medicine, Ehime University, 791-0295 Shitsukawa, Toon, Ehime, Japan

**Keywords:** Bone morphogenetic proteins, β-tricalcium phosphate, Rabbit, micro computed tomography, Histology

## Abstract

**Background:**

Bone morphogenetic proteins (BMPs) induce osteogenesis in various environments. However, when BMPs are used alone in the bone marrow environment, the maintenance of new bone formation is difficult owing to vigorous bone resorption. This is because BMPs stimulate the differentiation of not only osteoblast precursor cells but also osteoclast precursor cells. The present study aimed to induce and maintain new bone formation using the topical co-administration of recombinant human BMP-2 (rh-BMP-2) and zoledronate (ZOL) on beta-tricalcium phosphate (β-TCP) composite.

**Methods:**

β-TCP columns were impregnated with both rh-BMP-2 (30 µg) and ZOL (5 µg), rh-BMP-2 alone, or ZOL alone, and implanted into the left femur canal of New Zealand white rabbits (n = 56). The implanted β-TCP columns were harvested and evaluated at 3 and 6 weeks after implantation. These harvested β-TCP columns were evaluated radiologically using plane radiograph, and histologically using haematoxylin/eosin (H&E) and Masson’s trichrome (MT) staining. In addition, micro-computed tomography (CT) was performed for qualitative analysis of bone formation in each group (n = 7).

**Results:**

Tissue sections stained with H&E and MT dyes revealed that new bone formation inside the β-TCP composite was significantly greater in those impregnated with both rh-BMP-2 and ZOL than in those from the other experimental groups at 3 and 6 weeks after implantations (*p *< 0.05). Micro-CT data also demonstrated that the bone volume and the bone mineral density inside the β-TCP columns were significantly greater in those impregnated with both rh-BMP-2 and ZOL than in those from the other experimental groups at 3 and 6 weeks after implantations (*p *< 0.05).

**Conclusions:**

The topical co-administration of both rh-BMP-2 and ZOL on β-TCP composite promoted and maintained newly formed bone structure in the bone marrow environment.

## Background

Several clinical applications of recombinant human bone morphogenic proteins (rh-BMPs) have reportedly promoted new bone formation [1, 2]. BMPs act as signal transducers in the Smad signaling pathway to regulate mesenchymal stem cell differentiation during skeletal development, especially bone formation [3, 4]. For example, in orthopaedics surgery, rh-BMP has already been used to improve clinical results such as the novel operative technique of spinal fusion [5]. However, the use of rh-BMPs in certain orthopaedic surgeries performed in the intramedullary environment, e.g., total hip replacements involving large bone defects or intramedullary bone tumours, remains limited because more osteoclast progenitor cells are derived from hematopoietic stem cells in the bone marrow environment and rh-BMPs cannot achieve suitable osteogenesis inside of the bone marrow by promoting the differentiation of the osteoclast precursor cells, not only precursor cells which can be differentiated into osteoblast [6, 7]. In the intramedullary environment, it is difficult to achieve both bone formation and its maintenance.

To overcome these problems, we previously reported the effectiveness of the systemic administration of ZOL using rh-BMP-2/β-tricalcium phosphate (β-TCP) composite to promote the osteogenesis of newly formed bone in the bone marrow environment [8]. β-TCP has been reported as a good carrier for drug delivery of both rh-BMP and bisphosphonates to promote osteogenesis [9–12]. β-TCP, a bioactive bone substitute material, has high biocompatibility and good stability [13]. Moreover, ZOL has demonstrated to have a protective effect on bone tissue resorption by inhibiting the activity of osteoclasts at the local site [14, 15]. In the present study, we further investigated if the topical co-treatment of ZOL and the rh-BMP-2/β-TCP composite is useful in the promotion as well as the maintenance of new bone formation in the bone marrow environment. Should the intramedullary bone formation be achieved by only the topical administration of these drugs, this treatment may represent a safety and effective procedure to create bone formation in lesion sites, both from a clinical and morphological perspective.

In this study, the primary object was to achieve bone formation in the bone marrow environment and the secondary object was to maintain the formed bone tissue, by utilizing the combined effect of rh-BMP-2 in promoting bone formation and ZOL in maintaining bone tissue. In other words, we hypothesized that rh-BMP-2 could achieve bone formation in the bone marrow environment during the early treatment period and ZOL could maintain the newly formed bone tissue by inhibiting bone resorption for a certain period. The aim of this study was to investigate if the topical co-administration of rh-BMP-2/β-TCP/ZOL composite promoted osteogenesis and maintained the newly formed bone in the bone marrow environment.

## Materials and Methods

### Recombinant human BMP-2

This study used rh-BMP-2 produced in *Escherichia coli*, provided by Osteopharma, Inc (Osaka, Japan) [16]. Dimerization of the monomeric cytokine was obtained using published procedures [16, 17]. Rh-BMP-2 was reconstituted in sterile 0.01 N hydrochloric acid at 5 mg/mL and stored at 80℃ until use.

### Zoledronate

ZOL used in this study was purchased as a liquid solution as 4 mg/5 mL (Zometa™; Novartis Pharma K.K./ Tokyo, Japan) and stored at room temperature (approximately 25℃) until use. ZOL was diluted in phosphate-buffered saline (PBS, Wako, Osaka, Japan) to 5 µg ZOL per β-TCP column.

### β-TCP columns

β-TCP columns (diameter: 6 mm, length: 10 mm, porosity: 75 %) were manufactured and provided by HOYA (Tokyo, Japan), in a dry condition. The β-TCP columns were sterilized using dry heat (255 ℃, 3 h) and impregnated with each drug. The concentration of each drug was adjusted using 75 µL PBS per β-TCP column as follows: PBS only (Group 1), 30 µg of rh-BMP-2 (Group 2), 5 µg of ZOL (Group 3), or 30 µg of rh-BMP-2 and 5 µg of ZOL (Group 4). The drug lysates were infiltrated into the β-TCP columns in a laminar flow cabinet [8].

### Surgery and implantation of β-TCP columns

New Zealand white rabbits (n = 56 females, age: 18 to 20 weeks, body weight: 3.0–4.0 kg) were purchased from Japan SLC Co. (Shizuoka, Japan). All animals were acclimatized in cages with free access to food and water for 2 weeks. The β-TCP columns were surgically inserted into the medullary cavity at the distal position of the left femurs based on our previously described procedure [8]. Briefly, animals were anesthetized with a subcutaneous injection of ketamine (30 mg/kg body weight) and xylazine (10 mg/kg body weight). After exposure, the distal femur was reamed with a 6.2 mm hand drill to create a hole, a radiograph was taken for confirmation, and then a β-TCP column was inserted into the medullary cavity. During the postoperative period, all animals were maintained in cages (one rabbit per cage) in a temperature-controlled room (25℃) with ad libitum access to food and water and unrestricted movement at the animal care centre at our institution. At 3 and 6 weeks after the surgery, animals were sacrificed by intravenous injection of 100 mg/kg pentobarbital (Somnopentyl™, Kyoritu seiyaku, Tokyo, Japan) and the distal femurs containing the β-TCP were harvested. Seven rabbits were sacrificed in each group at the each timepoint. Harvested femurs were fixed in 4 % paraformaldehyde phosphate buffer overnight at 4℃ and stored in 70 % ethanol solution at 4℃ until use. No animals were excluded from experimental analysis. To reduce confounding factors as much as possible, the order of implanting β-TCP in each group was selected randomly. In the post-operative management, one cage was used for one animal and its locations in the animal care room were randomly selected at regular intervals for unification of environment. After surgery, the surgical wound condition, food intake, and activity were monitored and confirmed to be clear.

### Plane radiographs

Plane radiographs of the lateral views of the distal femurs were taken under anesthetization during the implantation surgery (0 weeks) and at 3 and 6 weeks after the surgery. Radiographs were obtained using a KXO-15ER apparatus (Toshiba Medical, Tochigi, Japan) at 50 kV and 100 mA for 0.08 s, and visualized using an FCR CAPSULA- 2V1 system (Fujifilm, Tokyo, Japan).

### Histological examination

Prior to histological evaluation, the fixed specimens were decalcified in 0.5 mol/L ethylenediaminetetraacetic acid (EDTA) solution (Wako, Osaka, Japan) for 2 weeks, dehydrated in a graded ethanol series (70 %, 80 %, 90 %, and 100 % ethanol), and embedded in paraffin wax. Mid-sagittal (longitudinal, along the implant) sections were cut into 4 µm slices in each plane. After preparation, the tissue sections were stained using haematoxylin/eosin (H&E) staining and Masson’s trichrome (MT) staining. New bone formation within the β-TCP columns was histologically assessed using previously described procedures with minor modifications [18]. Briefly, three high-powered fields (objective lens 20×) were randomly selected from three tissue sections from each the β-TCP column sample. The images were captured using a microscope with a built-in digital camera (DP 70; Olympus Corporation, Tokyo, Japan). Captured images were analysed using ImageJ™ software (National Institutes of Health, MD, USA). A total of 9 images captured in each group were analysed. The threshold for the measurement of the newly formed bone was set between 150 and 180 of the red channel in the software. New bone area (%) was estimated as the detected area/total area ×100 in each section. These new bone areas in the H&E and MT sections were defined as the primary outcomes in this study.

### Micro‐computed tomography

The implanted β-TCP columns were evaluated by micro-computed tomography (µ-CT) using an Aloka Latheta LCT200 (HITACHI, Tokyo, Japan) based on the previous published procedure [19, 20]. Briefly, the following conditions were maintained per image: slice width of 30 µm, voxel size of 30 × 120 µm, voltage of 80 kVp, and current of 50 µA. The area of β-TCP measurements was determined and the quality as bone in its area were quantitatively assessed using LaTheta software (version 2.10, Aloka). Bone volume/ total tissue volume (BV/TV) and bone mineral density (BMD) were evaluated according manufactural instruments. All sections were analysed by µ-CT (n = 7 in each group at 3 and 6 weeks after implantation). These quantitative bone assessments of µ-CT were defined as the secondary outcomes in this study.

### Ethical considerations

This study was approved by the Animal Research Committees of our institution (approval number 13,017). All applicable international, national, and/or institutional guidelines for the care and use of animals were followed. All procedures performed were in accordance with the ethical standards of the institution at which the study was conducted. This paper does not contain any studies with human participants performed by any of the authors.

### Statistical analysis

The results are presented as median and range (minimum and maximum). All variables were confirmed as parametric using the Kolmogorov-Smirnov test. The differences between groups were analysed using a one-way analysis of variance with Bonferroni’s multiple comparison test. To determine the adequate sample size, a power analysis was performed for the primary and secondary outcomes. According to a previous report on new bone area and the quantitative bone assessment of µ-CT, the expected differences in primary and secondary outcomes were 10 ± 5.5 % and 6 ± 3.5 %, respectively [8, 18]. Based on these findings, to provide an appropriate power (β = 0.80) with the significance level set at 0.05, a sample size of five cases or more was adequate to achieve the primary outcome and a sample size of six cases or more was adequate to achieve the secondary outcomes. Statistical significance was set at P < 0.05. Statistical analyses were performed using SPSS software, version 22 (IBM, NY, USA).

## Results

There were no statistically significant differences in body weight among each group at 0, 3, and 6 weeks after implantation (p = 0.63). The median and range of body weight (kg) at each time point for the groups 1, 2, 3, and 4 were as follows: 3.2 (2.9 to 3.3), 3.3 (3.0 to 3.4), 3.2 (3.0 to 3.3), and 3.2 (3.0 to 3.3) at 0 weeks, 3.3 (3.1 to 3.4), 3.2 (3.0 to 3.4), 3.2 (3.0 to 3.4), and 3.1 (3.0 to 3.3) at 3 weeks, 3.2 (3.0 to 3.3), 3.2 (3.0 to 3.5), 3.2 (3.1 to 3.3), and 3.0 (2.9 to 3.4) at 6 weeks, respectively. No cases of animals dropping out from observation during the study periods due to death or any other reasons were reported. Moreover, there was no complications, such as poor wound healing, after surgery.

### Macroscopy of implanted β-TCP columns in femoral bone marrow

**Fig. 1:**
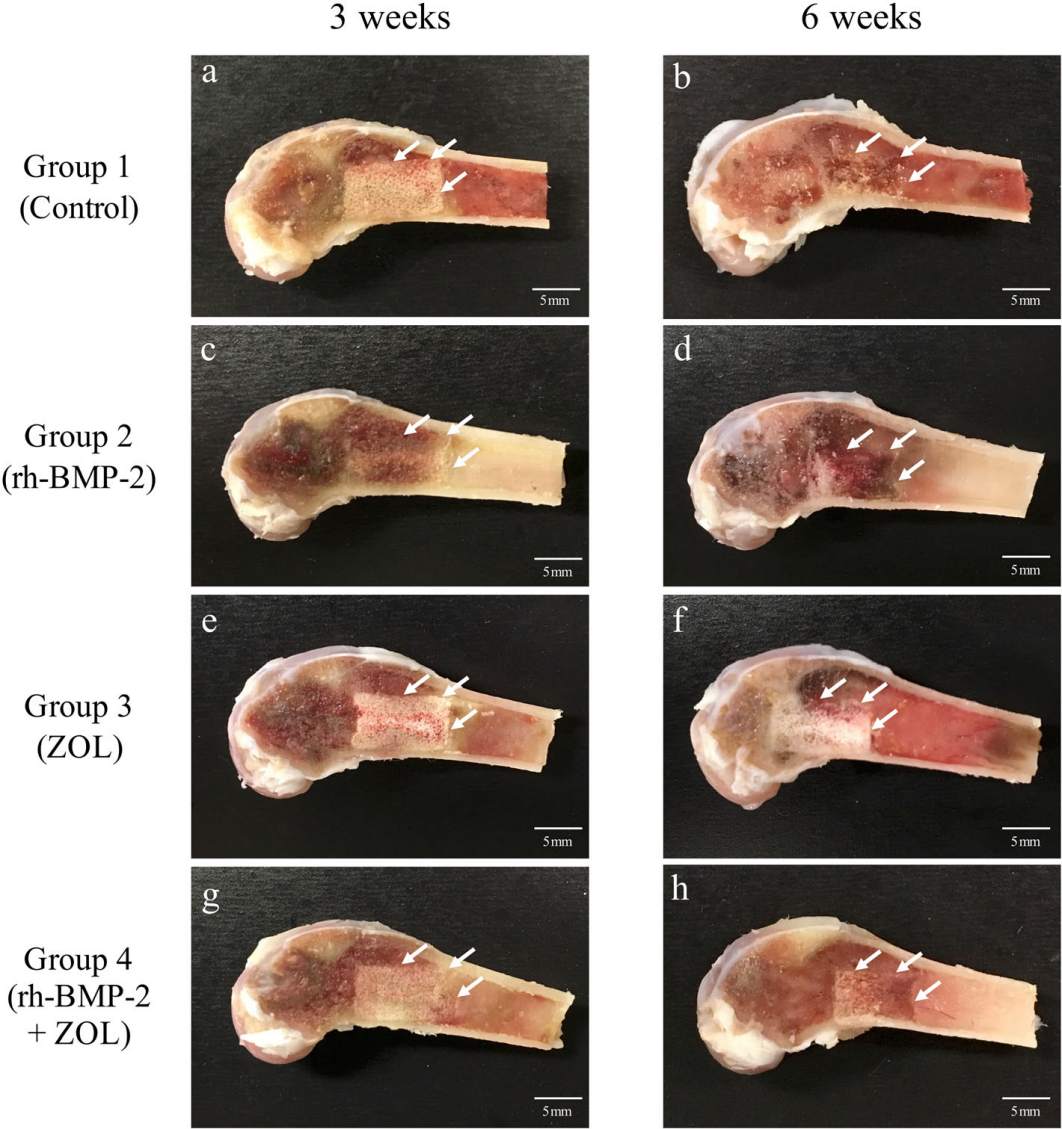
Representative photos of the left distal femurs of rabbits cut in the sagittal plane at 3 and 6 weeks after implantation. In the images, the left side is distal side of femur and the upper side is dorsal side of femur. Arrows point to a section of the edge of the implanted β-TCP. The gross appearances of implanted β-TCP columns gradually disappeared (**a**). The gross appearances of β-TCP columns in groups containing ZOL (f and h) were comparatively more recognizable than in the other groups without ZOL (**b** and **d**) at 6 weeks after implantation

At 3 weeks after implantation, the gross appearance of the implanted β-TCP column disappeared significantly in Group 2 (rh-BMP-2 alone) (Fig. 1c, d). However, in groups containing ZOL (Group 3 and 4), the β-TCP column remained recognizable at 6 weeks after implantation (Fig. 1e-h).

### Radiographic evaluations of implanted β-TCP columns in femoral bone marrow

**Fig. 2:**
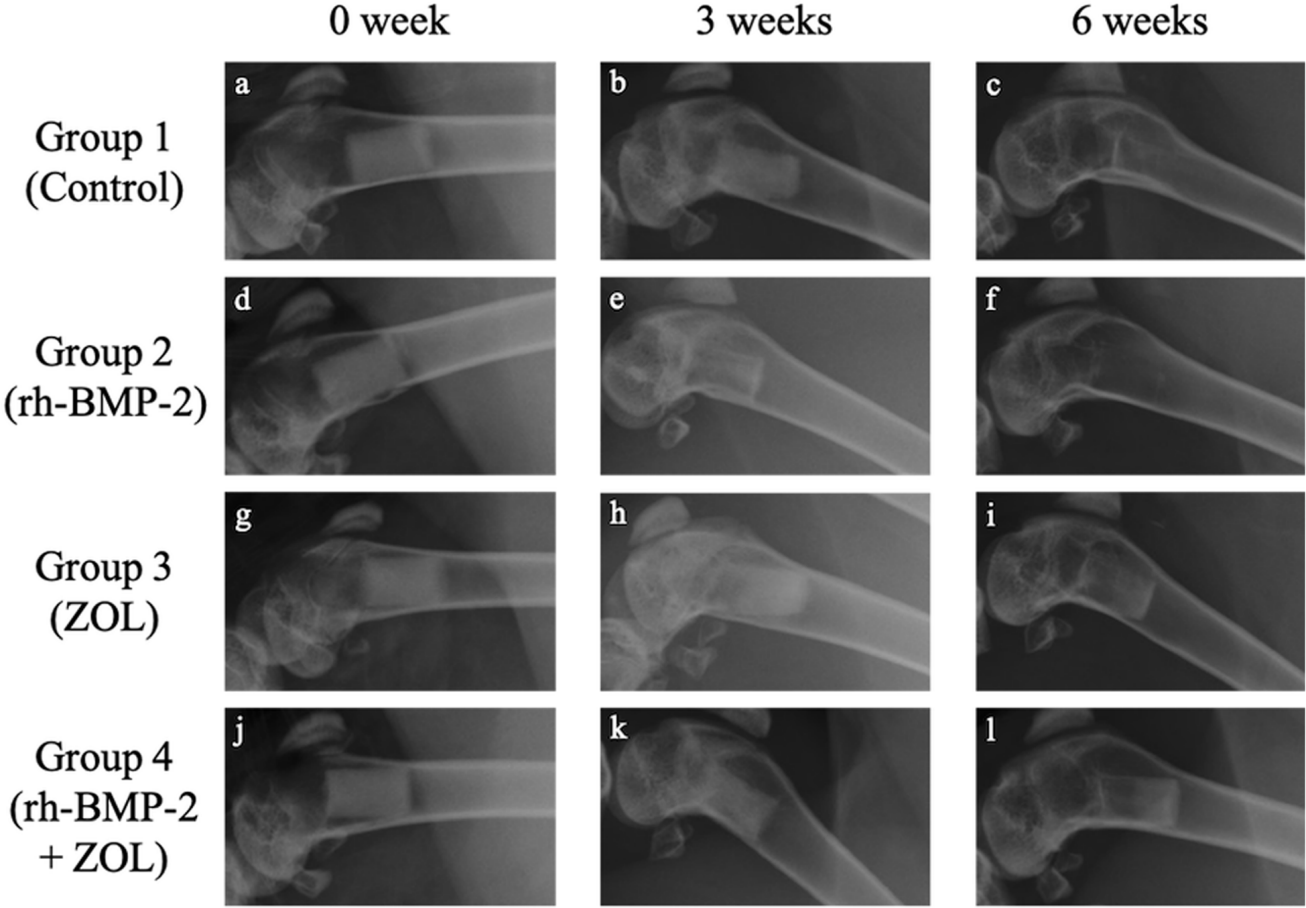
Representative X-ray images of the left distal femurs of rabbits from each group at 0, 3, and 6 weeks after implantation. In the images, the left side is distal side of femur and the upper side is dorsal side of femur. The radiolucencies at the area of implanted β-TCP columns gradually increased. The radiolucencies of β-TCP columns in groups containing ZOL (**i** and **l**) were comparatively more recognizable than in the other groups without ZOL (**c** and **f**) at 6 weeks after implantation

The X-ray images showed that the radiolucency inside the implanted β-TCP column tended to increase gradually in all groups (Fig. 2). However, in combination with the macroscopy analysis, the radiolucency inside of the β-TCP columns was comparatively suppressed in the ZOL-treated groups (Group 3 and 4) (Fig. 2g-l).

### Promotion and maintenance of bone formation in the bone marrow environment

**Fig. 3:**
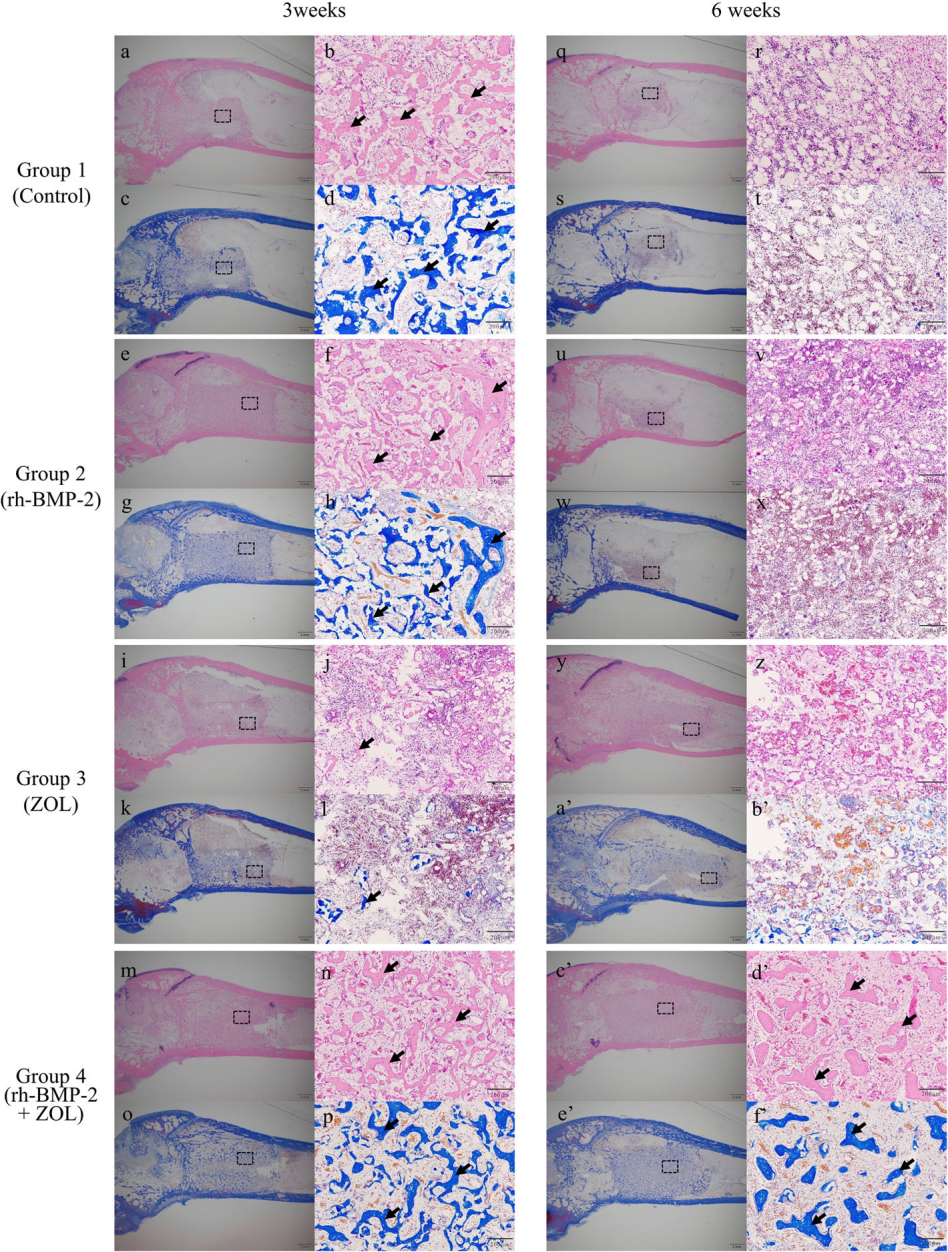
Representative H&E and Masson’s Trichrome stained sections of the left distal femurs of rabbits cut in the sagittal plane in each group at 3 and 6 weeks after implantation. In each image, the proximal section is displayed on the right and the dorsal section is displayed on the upper parts of the figure. The dotted box in the low-powered view (2×) indicates the range of high-powered view. The high-powered views (20×) were captured randomly from inside the implanted β-TCP areas for quantitative evaluation. The uniformly-stained tissue area, pointed by arrows, indicate newly formed trabecular bone structure. At 3 weeks after implantation, stained tissue areas were recognized as new bone area was significantly increased in groups containing rh-BMP-2 (**f**, **h**,**n**, and **p**). New bone area only remained in groups treated with both rh-BMP-2 and ZOL (d’ and f’) at 6 weeks after implantation. *Note*: H&E, Hematoxylin-Eosin; rh-BMP-2, recombinant human bone morphogenetic protein 2; ZOL, zoledronate; β-TCP, β-tricalcium phosphate

**Fig. 4:**
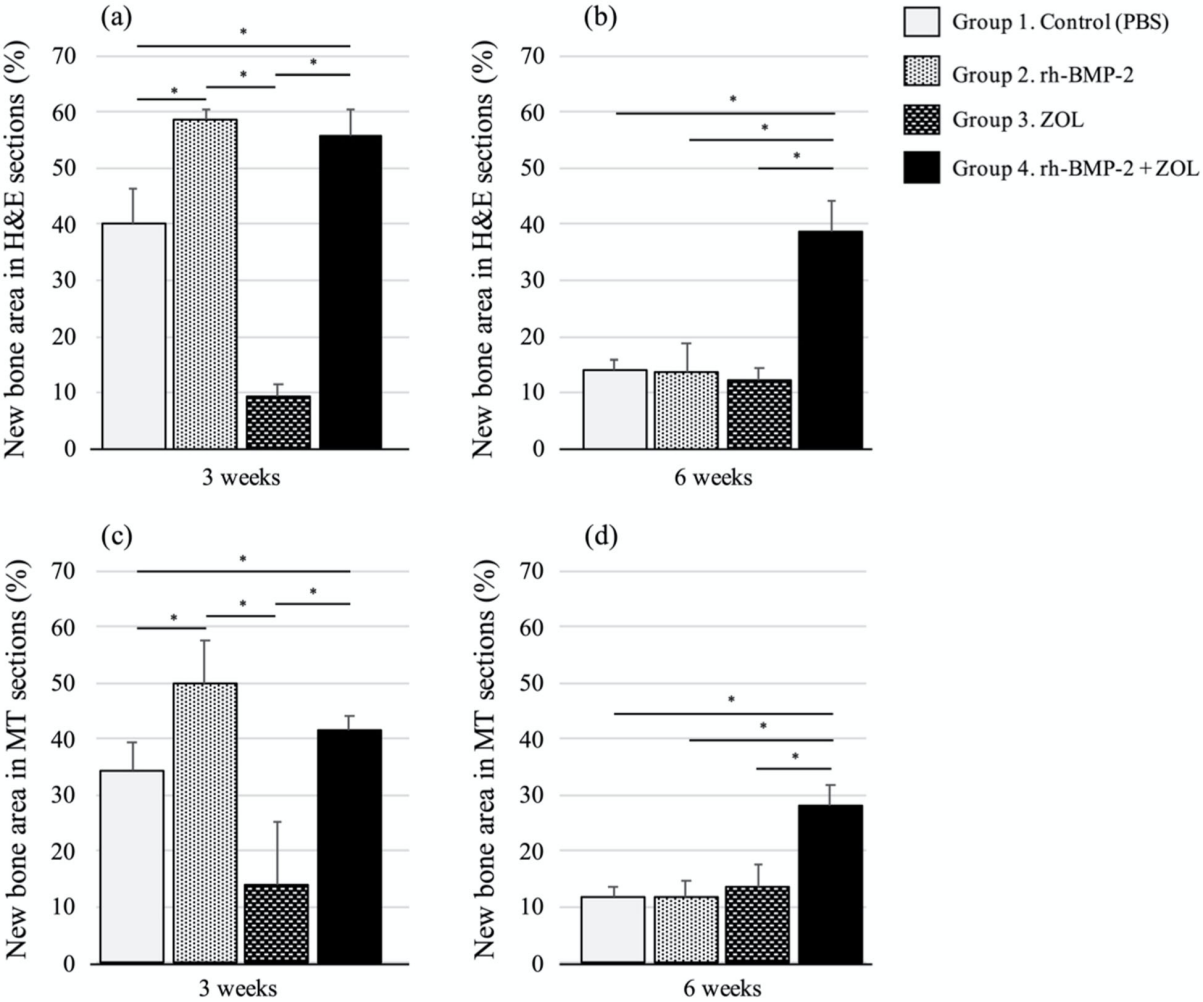
Quantitative evaluation of H&E sections and Masson’s Trichrome sections of the of the left distal femurs of rabbits in each group at 3 and 6 weeks after implantation. The columns and bars represent the means and standard deviations (n = 7), respectively. At 3 weeks after implantation, the groups containing rh-BMP-2 (Group 2 or 4) showed greater areas of new bone formation than the other groups (P < 0.05). However, at 6 weeks after implantation, only the group (Group 4) that involved the combination usage of both rh-BMP-2 and ZOL still showed areas of newly formed bone (P < 0.05). *: P < 0.05. Statistical differences between groups were determined using a one-way analysis of variance with Bonferroni’s multiple comparison test. *Note*: H&E, Hematoxylin-Eosin; MT, Masson Trichrome; rh-BMP-2, recombinant human bone morphogenetic protein 2; ZOL, zoledronate

Representative H&E and MT stained sections of tissues and their quantitative evaluations are shown in Figs. 3 and 4. At 3 weeks after implantation, the newly formed area of bone structure was significantly larger in the groups with rh-BMP-2 (Group 2 and 4) than in the groups without rh-BMP-2 (Group 1 and 3) (*p *< 0.001, Fig. 4a, c). Details of the statistical analysis of each parameter are as follows: group 1 vs. 2: *p *< 0.001 in H&E and *p *< 0.001 in MT; group 1 vs. 3: *p *= 0.05 in H&E and *p *= 0.06 in MT; group 1 vs. 4: *p *< 0.001 in H&E and *p *= 0.04 in MT; group 2 vs. 3: *p *< 0.001 in H&E and *p *< 0.001 in MT; group 2 vs. 4: *p *= 1.0 in H&E and *p *= 0.06 in MT; and group 3 vs. 4: *p *< 0.001 in H&E and *p* < 0.001 in MT. At 6 weeks after implantation, the newly formed area of bone structure in the group containing both rh-BMP-2 and ZOL (Group 4) was significantly larger than that in the other groups (*p *< 0.001, Fig. 4b, d). Details of the statistical analysis of each parameter are as follows: group 1 vs. 2: *p *= 1.0 in H&E and *p *= 1.0 in MT; group 1 vs. 3: *p *= 1.0 in H&E and *p *= 1.0 in MT; group 1 vs. 4: *p *< 0.001 in H&E and *p *= 0.03 in MT; group 2 vs. 3: *p *= 1.0 in H&E and *p *= 1.0 in MT; group 2 vs. 4: *p *< 0.001 in H&E and *p *< 0.001 in MT; and group 3 vs. 4: *p *< 0.001 in H&E and *p *= 0.006 in MT. The newly formed bone structure area in the Groups 1, 2, and 3 had almost disappeared at 6 weeks after implantation (Fig. 3q-z, a’, and b’). The actual values of new bone structure area in H&E and MT sections at 3 and 6 weeks after implantations are shown in Table 1.

**Table 1:** Histological assessments of new bone area in the marrow of a rabbit femur

	*Group 1(Control)*	*Group 2(rh-BMP-2)*	*Group 3(ZOL)*	*Group 4(rh-BMP-2 + ZOL)*	*p value*
**Haematoxylin and eosin (%, n = 9)**					
3 weeks after implantation	40.0(33.1 to 42.9)	56.9(40.9 to 66.9)	11.0(8.2 to 13.4)	57.9(40.2 to 68.6)	<0.001
6 weeks after implantation	13.9(11.1 to 18.0)	13.4(9.9 to 16.9)	12.3(9.9 to 15.6)	39.0(32.0 to 47.0)	<0.001
**Masson’s trichrome (%, n = 9)**					
3 weeks after implantation	36.5(25.4 to 40.9)	51.0(37.6 to 59.4)	13.5(10.7 to 19.9)	40.9(31.0 to 63.1)	<0.001
6 weeks after implantation	12.0(9.5 to 14.4)	11.9(6.9 to 15.5)	12.3(9.5 to 14.4)	30.0(24.3 to 30.1)	<0.001

Variables present percentages of new bone areas in tissues as median, minimum, and maximum. *P *values indicate the statistical differences between the groups. Note: *β-TCP *Beta-tricalcium phosphate, *rh-BMP-2 *Recombinant human bone morphogenetic protein-2, *ZOL *Zoledronate.

### Qualitative improvement of formed bone by topical co‐administration of rh-BMP2 and ZOL

**Fig. 5:**
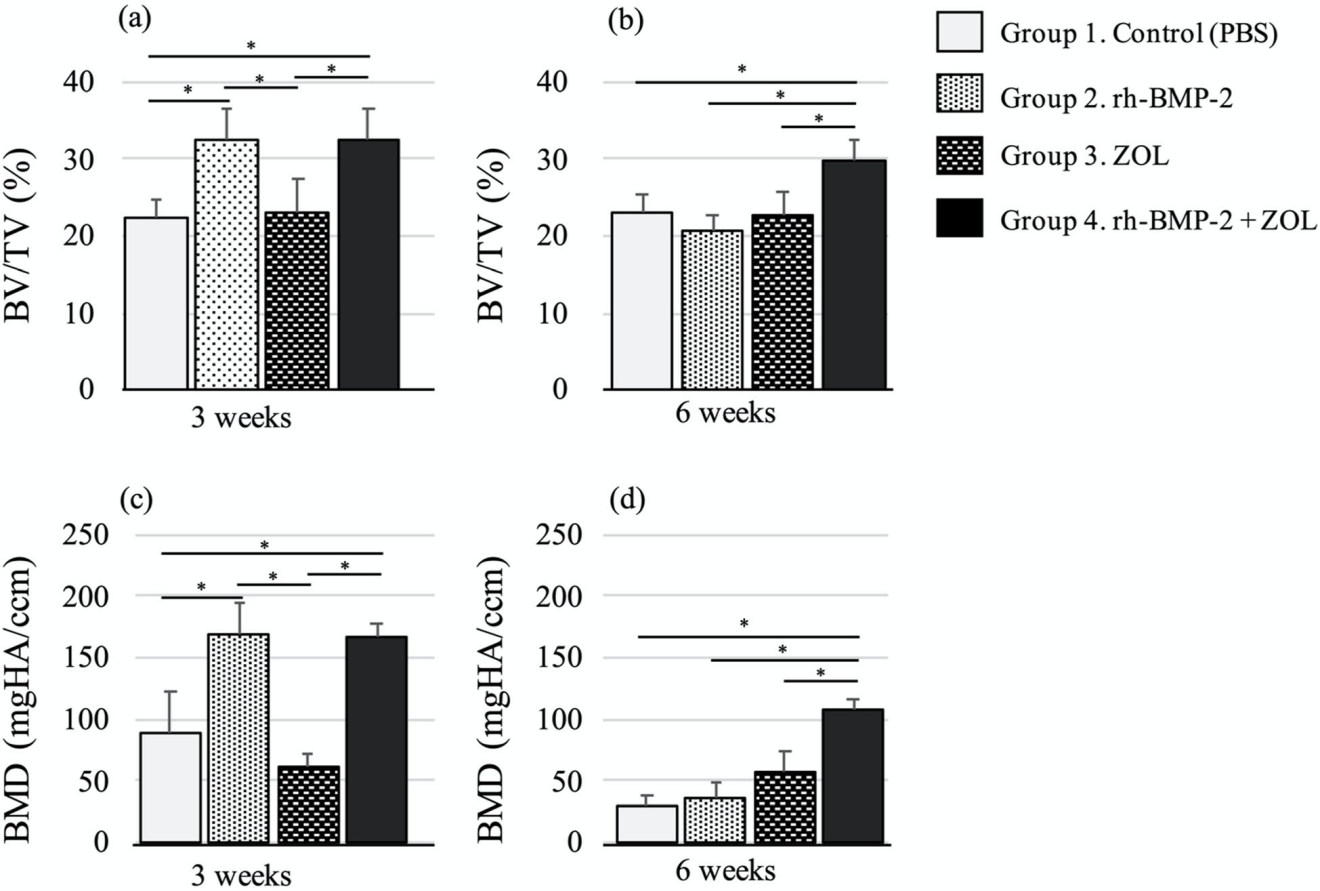
μ-CT evaluation of BV/TV and BMD in retrieved β-TCP implants at 3 and 6 weeks after implantation. The columns and bars represent the means and standard deviations (n = 7), respectively. *: *P* < 0.05. Statistical differences between groups were determined with the one-way ANOVA and post-hoc Bonferroni test. *Note*: BV/TV, Bone volume/ Total tissue volume; BMD, Bone mineral density

The qualitative differences of newly formed bone inside the implanted β-TCP columns between the groups were evaluated by µ-CT, and the results are shown in bar graphs in Fig. 5. At 3 weeks after implantation, groups with rh-BMP-2 (Group 2 and 4) showed significantly greater BV/TV and BMD than groups without rh-BMP-2 (Group 1 and 3) (*p *< 0.05, Fig. 5a, c). At 6 weeks after implantation, only the treatment group with both rh-BMP-2 and ZOL (Group 4) showed significantly greater BV/TV and BMD values than the other groups (*p *< 0.05, Fig. 5b, d). The actual values of BV/TV and BMD at 3 and 6 weeks after implantation are shown in Table 2.

**Table 2:** Quantitatively assessments of implanted β-TCP using μ-CT

	Group 1(Control)	Group 2(rh-BMP-2)	Group 3(ZOL)	Group 4(rh-BMP-2 + ZOL)	*p* value
**BV/ TV (%, n = 7)**					
3 weeks after implantation	21.7(17.8 to 27.1)	34.6(28.4 to 36.5)	24.3(20.2 to 28.5)	35.3(27.4 to 37.8)	<0.001
6 weeks after implantation	23.5(21.4 to 25.0)	22.8(20.0 to 25.1)	23.0(22.5 to 30.3)	30.4(27.0 to 33.9)	<0.001
**BMD (mgHA/ccm, n = 7)**					
3 weeks after implantation	96.0(66.3 to 104.8)	161.5(101.2 to 201.1)	70.2(48.1 to 80.2)	170.4(102.2 to 178.2)	<0.001
6 weeks after implantation	35.4(17.7 to 60.0)	40.0(23.8 to 52.4)	57.9(43.7 to 74.4)	109.1(80.2 to 115.4)	<0.001

Median, minimum, and maximum are provided. *P* values indicate the statistical differences between the groups. Note: *β-TCP* Beta-tricalcium phosphate, *rh-BMP-2 *Recombinant human bone morphogenetic protein-2, *ZOL *Zoledronate.

## Discussion

BMPs can induce osteogenesis by stimulating osteoblast differentiation [2], however, BMPs can also promote the catabolic activity of osteoclast [6, 21], which complicates the formation of bone in the bone marrow area. In this study, the radiological and histological parameters indicated that rh-BMP-2 promoted significantly osteogenesis in the bone marrow environment at 3 weeks after implantation. However, even though bone formation was achieved once at 3 weeks after implantation, the bone tissues gradually resorbed until 6 weeks after implantation due to the osteoclasts that were concurrently induced by rh-BMP-2 in the bone marrow environment [6]. We previously investigated the effect of ZOL on new bone formation induced by rh-BMP-2 in bone extramedullary and intramedullary environments. Systemic ZOL treatment via the rh-BMP-2/β-TCP composite was shown to promote and maintain new bone formation in bone marrow environment [8]. Local co-administration of ZOL via the rh-BMP-2/β-TCP composite has also been shown to promote and maintain new bone formation in the extramedullary environment for a long period of time [12]. In the present study, we aimed to clarify if the topical co-administration of ZOL was also effective in promoting and maintaining new bone formation induced by rh-BMP-2 in the bone marrow environment.

The ultimate goal of tissue regeneration engineering in the orthopaedic field is the accurate and effective formation of tissue at the necessary site. Therefore, we investigated whether the topical co-administration of ZOL and rh-BMP-2 would represent a useful procedure to facilitate and maintain bone formation in the bone marrow environment. As seen in systemic ZOL treatment, the topical treatment of ZOL co-administration with rh-BMP-2 also promoted and maintained new bone formation in the bone marrow environment. Topical administration of ZOL has been considered to reduce the associated side effects and limit the effect to a target site [22]. ZOL is known to cause side effects such as hypocalcemia, renal failure, or osteonecrosis of the jaw. Therefore, the topical administration of ZOL can be effective in patients in whom systemic administration is inappropriate due to side effects [23]. A systematic review showed that β-TCP is one of the most commonly used biocompatible materials [13]. β-TCP has high biocompatibility and is an ideal material for clinical application [24, 25]. It has been shown to be effective in bone conduction on its own, but it is also often used as a carrier of some drugs to accelerate effectiveness [13]. It has been reported that β-TCP as a carrier for the local administration of both rh-BMP-2 and ZOL is useful for new ectopic bone formation [12], and our findings further demonstrated that β-TCP is a useful carrier of rh-BMP-2 and ZOL for effective bone induction in the bone marrow environment.

Bone formation in the bone marrow environment by local drug administration is clinically important because it leads to the development of biomaterials for surgical implants in the medullary cavity, such as intramedullary nails and the femoral stem of total hip replacements. Moreover, these biomaterials can offer novel therapeutic substitutes that can be used for the regeneration of bone cavities after the surgical removal of bone tumors, osteonecrosis lesions, or vertebral fractures. Local administrated of ZOL has been shown to directly suppress the bone resorption action of the osteoclasts in the local area [26]. The local effects of ZOL may also enable complications related to systemic bisphosphonate therapy, such as renal disorders or osteonecrosis of the jaw, to be avoided [27]. Therefore, β-TCP material treated with a combination of rh-BMP-2 and ZOL was shown to effectively promote and maintain bone formation in the bone marrow environment.

This study contains a few limitations, e.g., a single animal model and a single dose of therapeutic agents was used. Future studies should be conducted to assess the underlying detailed molecular mechanisms of the combination therapy that produced the observed therapeutic effect.

## Conclusions

In summary, the combination of locally administered rh-BMP-2 and ZOL via β-TCP column materials promoted new bone formation in the bone marrow and enabled the maintenance of the newly formed bone for 6 weeks after implantation. Our findings may contribute to the development of the orthopaedic field, especially involving clinical approaches for cases that require bone regeneration in the bone marrow environment.

## Data Availability

The datasets used and/or analyzed during this study are available from the corresponding author on reasonable request.

## Abbreviations

BMPs: Bone morphogenetic proteins
rh: Recombinant human
ZOL: Zoledronate
β-TCP: Beta-tricalcium phosphate
H&E: Haematoxylin/eosin
MT: Masson’s trichrome
CT: Micro-computed tomography
PBS: Phosphate-buffered saline
EDTA: Ethylenediaminetetraacetic acid
BV/TV: Bone volume/ total tissue volume
BMD: Bone mineral density
